# Antiinflammatory photodynamic therapy potential of polyoxyethylene-substituted perylene diimide, nitrocatechol, and azo dye

**DOI:** 10.3906/kim-2104-8

**Published:** 2021-06-16

**Authors:** Özgül HAKLI, Kasım OCAKOĞLU, Furkan AYAZ

**Affiliations:** 1 Department of Chemistry, Faculty of Science, Muğla Sıtkı Koçman University, Muğla, Turkey; 2 Department of Engineering Fundamental Sciences, Faculty of Engineering, Tarsus University, Mersin, Turkey; 3 Department of Biotechnology, Faculty of Arts and Science, Mersin University, Mersin, Turkey

**Keywords:** Inflammation, perylene, photodynamic therapy, macrophages, antiinflammatory compounds

## Abstract

Photodynamic therapy (PDT) applications enable light-controlled activation of drug candidates instead of their constitutive activities to prevent undesired side effects associated with their constant activities. A specific wavelength of light is utilized to enable electron mobility in the chemical structure, which results in differential activities that may alter cell viability and cellular functions. Canonical photodynamic therapy applications mostly focus on cytotoxicity-based antimicrobial and anticancer properties of the PDT agents. In this study, we focused on subtoxic concentrations of three different molecules containing polyoxyethylene group and examined their antiinflammatory activities on stimulated mammalian macrophages. Stimulated macrophages produce proinflammatory cytokines TNF and IL6. In the presence of a light source, our PDT agents were activated for 5 and 10 min during their application to the macrophages. Based on the ELISA results, the compounds had anti-inflammatory PDT activities. Trypan blue staining results suggest that these derivatives exerted their activities without leading to cytotoxicity. Our results suggest noncanonical PDT applications of these derivatives that can alter cellular activities without leading to cell death.

## 1. Introduction

**Figure 1:**
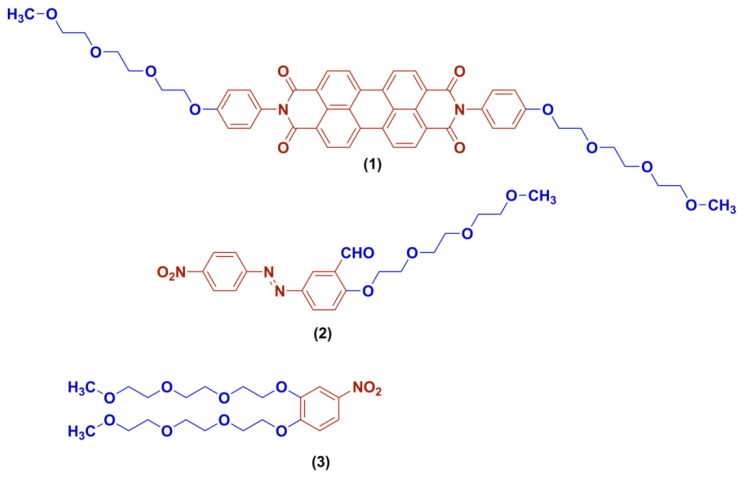
Molecular structures of the compounds containing polyoxyethylene group.

Polyoxyethylene side chains are preferred in molecules for various reasons due to their greater conformational flexibility, increased polarity, and increased solubility [1]. The molecules described here possess polyoxyethylene substituent groups (Figure 1). Perylene diimide derivative **1** was synthesized by using the triethylene glycol monomethyl ether derivative polyoxy group substituted on a benzene ring. The polyoxyethylene side chain increases the polarity and solubility of the molecule. Perylene-3,4,9,10-tetracarboxylic diimide derivatives containing a π-conjugated system with a perylene aromatic core have gathered a substantial interest in the field as fluorophores. Both symmetrical and unsymmetrical perylene diimides (PDIs) have been chemically and photodynamically stable, are easy to synthesize and characterize, have high solubility in organic solvents, have convenient excitation and emission spectra in the visible region (400–450 nm B band, 500–700 nm Q band). PDIs have been widely used in broad areas. They have versatile electrical and optical utilizations. These are not limited but can be listed as semiconductors in photovoltaic systems, electrophotographic technologies, dye lasers, transistors, light-emitting diodes, and photorefractive thin films [2–5]. They have been also used in cancer theranostics due to their high thermostability, large π-π conjugated structure, superior photochemical properties, and high fluorescence quantum yields [6]. These molecules have been used as ligands for photodynamic therapy applications. They are known to inhibit the telomerase activity in cancer cells. These molecules’ mechanism of action on cancer cells is through the production of reactive oxygen species upon receiving the visible light irradiation. These molecules’ binding ability to G-quadruplex structures of the DNA has been an active area of investigation since this DNA structure is well known for telomerase activity. By targeting this region, telomerase activity and, therefore, cancer cell proliferation will be eliminated [7]. Although active oxygen species-based functional properties of PDIs as a PDT agent have been studied extensively, their subtoxic and noncanonical PDT potentials have not been investigated yet [8–12]. Azo compound (molecule **2**) containing polyoxy group was obtained by using a chlorine derivative of triethylene glycol and aniline derivative. The polyoxyethylene group as a substituent was bonded to the oxygen atom in the hydroxyl group in the benzene ring. Azo dyes contain the (−N = N−) part in their structure. This structure is conjugated with two, different or identical, mono- or polycyclic aromatic systems. They have found wide application in the pharmaceutical, cosmetic, food, dyeing/textile industry, and analytical chemistry due to their specific physicochemical properties and biological activities. There are numerous biological activities that make them medically attractive compounds [13–14]. Molecule **3** containing polyoxyethylene units was obtained by using chlorine derivative of triethylene glycol and 4-nitro catechol. Polyoxyethylene units are attached to the oxygen atom in both hydroxyl groups as substituents. 4-Nitrocatecol and other catechol derivatives are known to be active against the intestinal bacterial panel, and nitrophenol derivatives have antimicrobial activity [15].

In this study, we focused on subtoxic concentrations of PDI derivatives as potential PDT agents to suppress inflammatory responses created by mammalian macrophages. These PDI derivatives were chosen due to their photodynamic activity potentials, and, in this study, we aimed to investigate the differences in their efficiencies for their possible utilization in the future. Macrophages are chosen for this study due to their proinflammatory TNF, IL6, IL1, IL12, and GMCSF production potentials. They can regulate the immune system by these signaling molecules. Therefore, to decipher the activity of a possible immunomodulatory compound, initial screening on these cells gives crucial information. Moreover, these cells can phagocytose cell debris and infectious agents as well as their exo and endotoxins to further present them to the T cells of the adaptive immunity. Together with cytokine production, this presentation-based activation of the T cells makes macrophages an important player in the determination of the type and strength of the immune response against a certain danger to the body [16, 17].

Hence, to investigate the antiinflammatory PDT potentials of PDI derivatives, they were tested on activated mammalian macrophages. Based on TNF and IL6 ELISA results, these derivatives had antiinflammatory activities, and their PDT potential was differential based on structural differences.

## 2. Materials and methods

### 2.1. Synthesis of N,N’-Bis (4-{2-[2-(2-methoxyethoxy ethoxy]ethoxy} phenyl)-3,4:9,10-perylene tetracarboxydiimide (1)

The synthesis procedure was illustrated in our previous work [18, 19]. Perylene-3,4:9,10-tetracarboxylic acid bisanhydride (0.69 mmol, 270 mg), 4-{2-[2-(2-methoxyethoxy) ethoxy] ethoxy} aniline (1.5 mmol, 382.5 mg) and imidazole (5 g) were heated at 140 °C for 4.5 h under inert atmosphere. Then 2N HCl (200 mL) was added to the reaction solution, and the resulting mixture was stirred for 1 h at room temperature. It was extracted with chloroform (600 mL). The organic phase evaporated under vacuum, and crude product was purified by column chromatography (neutral alumina; CH_2_Cl_2_-MeOH, 10:1). Yield: 62%. FT–IR (cm^−1^): 2922-2867, 1704-1663, 1595-1512, 1455-1404, 1361, 1299-1255, 1178, 1124. ^1^H NMR (CDCl_3_, 400 mHz): δ (ppm) = 8.68-8.59 (q, 8H, Ar*H*),7.20-7.02 (q, 8H, Ar*H*), 4.15 (t, 4H, ArOC*H_2_*−), 3.84 (t, 4H, ArOC*H_2_*C*H_2_*), 3.7 (t, 4H, −OCH_2_–CH_2_OCH_3_), 3.63 3.61 (m, 8H, – OC*H_2_*C*H_2_*O−), 3.5 (t, 4H, −C*H_2_*OCH_3_), 3.33 (s, 6H, −OC*H_3_*), C_50_H_46_N_2_O_12_.

### 2.2. Synthesis of 2{2[2(2-methoxyethoxy)ethoxy]ethoxy}-5-[(E)-(4 nitrophenyl)diazenyl]benzaldehyde (2)

1-(3-formyl-4-hydroxyphenylazo)-4-nitrobenzene (2) was synthesized before according to literature [20,21]. Chloro-2-[2-(2-methoxyethoxy)ethoxy]ethane (2.8 mmol) was added to a mixture of 1-(3-formyl-4-hydroxy-phenylazo)-4 nitrobenzene (6.4mmol) and K_2_CO_3_ (12.8 mmol) in DMF (20mL) under inert atmosphere. The mixture was heated at 140 °C for 19h and then refluxed for 2h. After concentrating, purification was done by column chromatography (Silica, CHCl_3_:MeOH, 10:0.5). Yield: 85%. ^1^H NMR (CDCl_3_, 400 mHz): δ (ppm) = 10.05 (s, 1H), 8.39(d, 2H), 8.27(t, 1H), 8.22(t, 1H), 8.01 (d, 2H), 7.14(d, 1H), 4.3(t, 2H), 3.9(t, 2H),3.7(t, 2H),3.6(m, 4H), 3.5(t, 2H),3.33(s, 3H).C_20_H_23_ N_3_O_7_.

### 2.3. Synthesis of 1,2-di{2-[2-(2-methoxyethoxy)ethoxy]ethoxy}-4-nitrobenzene(3)

The synthesis procedure was illustrated in our previous work [22]. Chloro-2-[2-(2-methoxyethoxy)ethoxy]ethane (2.34 g, 2.8 mmol) was added to a mixture of 4-nitrocatechol (1 g, 6.4 mmol) and K_2_CO_3_ (1.77 g, 12.8 mmol) in DMF (20 mL) under an inert atmosphere. The mixture was heated at 140 °C for 19 h by stirring. And then it was refluxed for 2 h. It was stopped after 2 h. After DMF was removed by rotary evaporation, the resulting solution was purified through a column. The eluent was 4% methanol in CHCl_3_. Yield: 85%. H^1^ NMR (CDCl_3_, 400 mHz): δ (ppm) = 7.8-6.9 (m, 3H, Ar-*H*), 4.2-4.1 (q, 4H, ArOC*H_2_*−), 3.88-3,85 (m, 4H, ArOCH_2_C*H_2_*−), 3.72-3,69 (m, 4H, −OC*H_2_*CH_2_OCH_3_), 3.64 3,58 (m, 8H, −OC*H_2_*C*H_2_*O−), 3.51-3,48 (m, 4H, CH_2_C*H_2_*OCH_3_), 3.33 (s, 6H, −OC*H_3_*); C_20_H_33_ NO_10_.

For the photodynamic activation Xenon Light 300 Watt/m^2^ was used.

### 2.4. Stimulation of mammalian macrophages to test the antiinflammatory PDT activities of compounds

These protocols were explained in detail in our previous studies [23–30]. Mammalian macrophages were RAW 264.7 mouse macrophage cells from ATCC. These cells were grown in RPMI 1640 media together with 10% fetal bovine serum, 1% antibiotics (100 μg/mL penicillin and 100 μg/mL streptomycin), and sodium pyruvate. Cell incubations were done in 37 °C 5% CO_2_ incubator [23,24,26–30,31]. 10^6^ macrophages were plated into each well of 24 well plates in 1 mL final volume. They were let adhere to the bottom of the plates after overnight incubation before their activation. For stimulations, sterile DMSO was added into the negative control wells, 1 ug/mL of LPS (1mg/mL, Enzo Life Sciences, Salmonella Minnesota R595), sterile DMSO was added into the positive control wells, and 1ug/mL and 10ug/mL of the compound’s molecules (Figure 1) were put into the appropriate wells with or without 1 ug/mL LPS. These conditions were created as triplicate setups: dark conditions, 5 min Xenon light exposure, and 10 min Xenon light exposure. Before 24 h of incubation, light exposure procedures were conducted right after the addition of the stimulants and compounds. Afterward, the plates were put into the incubator for 24 h. After the incubation period, the supernatants were collected and kept at −80 °C for ELISA. Trypan blue staining with a hemocytometer was done to assess the cell viability. TNFα and IL6 BD ELISA kits were purchased, and ELISA protocols were followed by using the manufacturer’s instructions to determine the changes in the production of these proinflammatory cytokines [23,24,26–30,32].

Statistical analysis: 3 biologically independent data sets were combined to do a student t-test by using GraphPad Prism Software 5 [23, 24, 26–30].

## 3. Results

### 3.1. Compounds lacked immunostimulatory activities on mammalian macrophages

**Figure 2:**
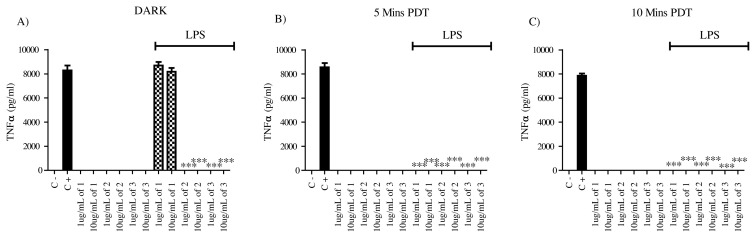
TNFα ELISA results for 1 ug/mL and 10 ug/mL compound (**1**), compound (**2**), and compound (**3**) application to either untreated or 1 ug/mL LPS activated macrophages. Supernatants were analyzed after 24 h of incubation, N = 3 (*p < 0.001, **p < 0.0005, ***p < 0.0001). The DMSO, as the solvent of the compounds, was added into the negative control and positive control wells in 10uL volume, 1 ug/mL LPS was used in positive control wells. This setup was used for dark conditions (A) as well as for 5 min (B) and 10 min (C) Xenon light exposure. The light exposure was done right after the addition of the compounds and afterward, the 24 h incubation was started.

**Figure 3:**
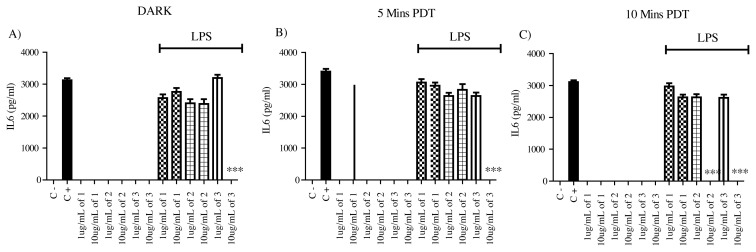
IL6 ELISA results for 1 ug/mL and 10 ug/mL compound (**1**), compound (**2**), and compound (**3**) application to either untreated or 1 ug/mL LPS activated macrophages. Supernatants were analyzed after 24 h of incubation, N = 3 (*p < 0.001, ** p < 0.0005, ***p < 0.0001). The DMSO, as the solvent of the compounds, was added into the negative control and positive control wells in 10uL volume, 1 ug/mL LPS was used in positive control wells. This setup was used for dark conditions (A) as well as for 5 min (B) and 10 min (C) Xenon light exposure. The light exposure was done right after the addition of the compounds and afterward, the 24 h incubation was started.

Compounds were tested on unactivated mammalian macrophages to determine their possible intrinsic stimulatory activities on unstimulated macrophages. They did not exert the production of proinflammatory TNF and IL6 cytokines by the macrophages in the absence of LPS as an activator. Moreover, having dark and light-induced conditions did not change the results, and these compounds lacked immunostimulatory activity under all conditions (Figure 2 and Figure3).

### 3.2. Compounds had differential antiinflammatory PDT potentials on activated mammalian macrophages based on TNF and IL6 production levels

In order to test the antiinflammatory potentials of compounds, they were also tested on mammalian macrophages in the presence of LPS as a stimulant. Under dark conditions other than compound **1**, all the derivatives had antiinflammatory activities on macrophages in terms of TNF production. TNF was not detected in the supernatants of the groups that were treated with compounds **2** and **3**, whereas compound **1** did not affect TNF production levels in dark conditions. Upon light-induced activation, compound 1 gained antiinflammatory activity and completely suppressed the production of TNF both after 5 min and 10 min of light exposure. Compound **2** and **3** retained their antiinflammatory activity similar to dark conditions based on TNF production. These results suggest that all the compounds have antiinflammatory activity on the activated macrophages by reducing the TNF production but compound **1** had PDT potential. Compounds **2** and **3** were constitutively active since they were able to suppress TNF production independent of light-induced activation (Figure 3).

IL6 was another cytokine that we measured from activated macrophages. Based on our results, compound **1** lacked the ability to alter IL6 production both in dark and light conditions. Whereas, compounds **2** and **3** gained a strong antiinflammatory activity only after light exposure. These compounds (**2** and **3**) could not change IL6 production by activated macrophages in dark conditions. Only their (**2** and **3**) higher concentrations showed PDT potential. Compound **3**’s higher concentrations lead to complete knockout of IL6 production by activated macrophages after 5 and 10 min of Xenon light exposure. Whereas, compound **2** gained this activity on IL6 production after 10 min of Xenon light exposure only with its high concentrations.

These results imply that compound **1** would not be useful in antiinflammatory PDT applications where IL6 production is a target, but it will be useful for targeting TNF while sparing IL6 production by macrophages. Compounds **2** and **3** can constitutively block TNF production independent of light exposure, but they exerted an effective antiinflammatory PDT potential to suppress IL6 production with their higher concentrations only after light exposure (Figure 2 and Figure3).

### 3.3. Compounds did not have cytotoxic effects on mammalian macrophages

In this study, we focused on nontoxic concentrations of these agents, and we aimed to determine their biological activities at subtoxic concentrations both at dark and light-induced conditions. Trypan blue staining was conducted to confirm that these compounds were nontoxic at the concentrations that they were used. Our results suggest that they did not have a substantial effect on the cell viability both in dark and light conditions.

## 4. Conclusion

Compounds have been studied for their photovoltaic applications in solar cells. These molecules also possess biological activities. Compounds exerted anticancer activity, and some studies also suggest their possible utilization for tumor cell imaging due to their ability to interact with DNA [9–12, 32]. Moreover, polyoxyethylene groups have been used as prodrug and drug delivery molecules in various applications. They increase the uptake of the drug molecules by the cells and tissues while enabling more controlled delivery of the main active ingredients of the drug formulations. Due to their biocompatible nature as well as the ability to increase the bioavailability and solubility of drug molecules, it was designed a perylene diimide derivative substituted with polyoxyethylene groups [33–37]. During the production process, we obtained two benzene derivatives with polyoxyethylene substitutions.

In this study, we examined these molecules’ antiinflammatory PDT potential on mammalian macrophages. Studies mostly focus on a cytotoxic aspect of PDT and present data on antitumor, antifungal, or antibacterial properties of PDT agents [38–44].

**Figure 4:**
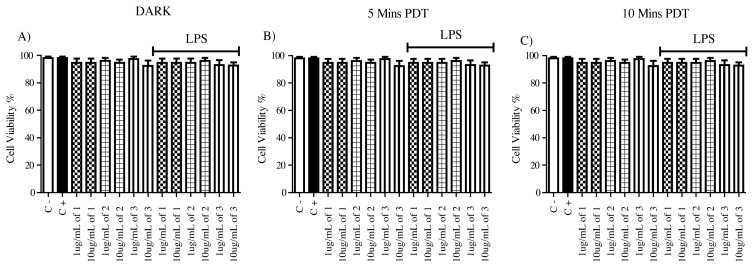
Cell viability results for 1 ug/mL and 10 ug/mL compound (**1**), compound (**2**), and compound (**3**) application to either untreated or 1 ug/mL LPS activated macrophages. The cell counting was done with Trypan blue after 24 h of incubation, N = 3 (*p < 0.001, **p < 0.0005, ***p < 0.0001). The DMSO, as the solvent of the compounds, was added into the negative control and positive control wells in 10uL volume, 1 ug/mL LPS was used in positive control wells. This setup was used for dark conditions (A) as well as for 5 min (B) and 10 min (C) Xenon light exposure. The light exposure was done right after the addition of the compounds and afterward, the 24 h incubation was started.

In this study, we utilized subtoxic concentrations of the agents, which were confirmed by Trypan Blue staining (Figure 4). Intermediate compounds had similar activities on TNF production levels of activated macrophages, and they were strong antiinflammatory agents even in dark conditions. They kept this property and knocked TNF production out by activated macrophages after 5 min and 10 min of light exposure as well. On the other hand, having perylene diimide in the structure enabled a PDT potential, since PDI derivative was inert on stimulated macrophages for the production of TNF in the dark and gained strong antiinflammatory property by completely knocking out TNF production after 5 min and 10 min of Xenon light exposure. These results suggest that having perylene diimide in the molecular formula enabled PDT application potential.

IL6 was another pro-inflammatory cytokine that we examined in this study. Our results suggest that these compounds were not able to alter IL6 production by stimulated macrophages, which could be an advantage in situations where TNF production should be suppressed while sparing IL6 production by the macrophages. Moreover, the intermediate products suppressed IL6 production at their higher concentrations especially with light exposure. Photodynamic therapy presents opportunities for more controlled and localized activation of drug molecules to increase their efficiency and decrease their possible side effects. So far, most of the studies utilize photosensitizers and different dye molecules as PDT agents, and they mostly focus on their cytotoxic capacities [38–44].

**Figure 5:**
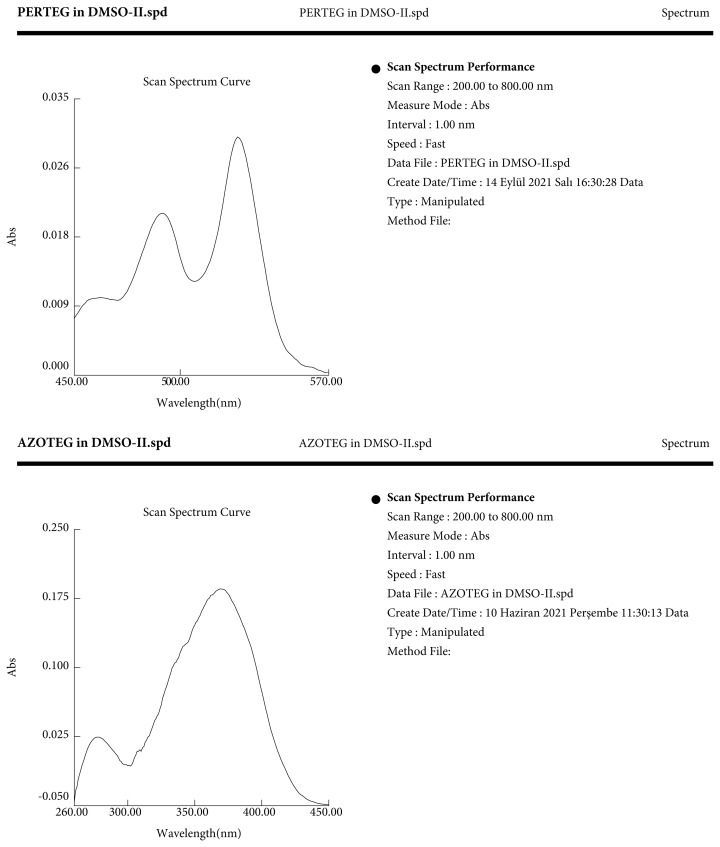

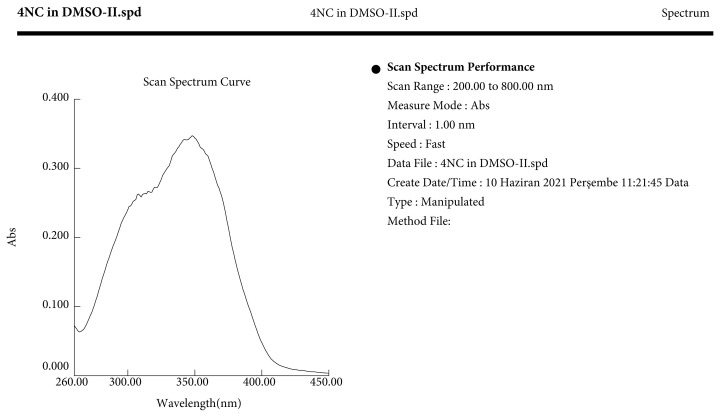
UV-Vis absorption spectra of compound (**1**), compound (**2**), and compound (**3**) in DMSO.

The uV spectrums of the studied compounds are given in Figure 5. Compound 1 has characteristic absorption peaks at 455 nm, 492 nm, and 527 nm corresponding to π– π* singlet transition. Compound 2 shows absorption peaks at 277 nm and 369 nm. Compound 3 gives absorption peaks at 302 nm and 348 nm.

Our results suggest that compound 1 was not as effective as compound 2 and 3 in terms of its antiinflammatory activities. The reason behind this difference might be due to the differences in their structures and how it might affect their interactions with the signaling pathways that play important role in the inflammatory response. In our future studies, we are planning to focus on their possible effects on inflammatory pathways such as JNK, ERK and PI3K [45].

In this study, we aimed to focus on their subtoxic concentrations and determine possible PDT activities. The compounds that we are presenting in this study had antiinflammatory PDT potential on activated mammalian macrophages. Further studies on their in vivo efficacy will bring out their potential utilization in the clinic to cope with inflammatory and autoimmune diseases.
